# The salivary scavenger and agglutinin binds MBL and regulates the lectin pathway of complement in solution and on surfaces

**DOI:** 10.3389/fimmu.2012.00205

**Published:** 2012-07-16

**Authors:** Martin P. Reichhardt, Vuokko Loimaranta, Steffen Thiel, Jukka Finne, Seppo Meri, Hanna Jarva

**Affiliations:** ^1^ Infection Biology Research Program, Department of Bacteriology and Immunology, Haartman Institute, University of Helsinki,Helsinki, Finland; ^2^ Department of Medical Biochemistry and Genetics, University of Turku,Turku, Finland; ^3^ Department of Biomedicine, University of Aarhus,Aarhus, Denmark; ^4^ Division of Biochemistry and Biotechnology, Department of Biosciences, University of Helsinki,Helsinki, Finland; ^5^ Helsinki University Central Hospital Laboratory,Helsinki, Finland

**Keywords:** complement regulation, gp340, lectin pathway, mannan-binding lectin, mucosal immunity, salivary agglutinin, scavenger receptor cysteine-rich

## Abstract

The salivary scavenger and agglutinin (SALSA), also known as gp340, salivary agglutinin and deleted in malignant brain tumor 1, is a 340-kDa glycoprotein expressed on mucosal surfaces and secreted into several body fluids. SALSA binds to a broad variety of microbes and endogenous ligands, such as complement factor C1q, surfactant proteins D and A, and IgA. Our search for novel ligands of SALSA by direct protein-interaction studies led to the identification of mannan-binding lectin (MBL) as a new binding partner. We observed that surface-associated SALSA activates complement via binding of MBL. On the other hand, soluble SALSA was found to inhibit *Candida albicans*-induced complement activation. Thus, SALSA has a dual complement activation modifying function. It activates the lectin pathway when bound to a surface and inhibits it when free in the fluid phase. These activities are mediated via a direct interaction with MBL. This suggests that SALSA could target the innate immune responses to certain microorganisms and simultaneously limit complement activation in the fluid phase.

## INTRODUCTION

The scavenger receptor cysteine-rich (SRCR) protein, known as gp340, salivary agglutinin (SAG) and deleted in malignant brain tumor 1 (DMBT1; GenBank accession no. BAA78577.1), is a 340-kDa glycoprotein expressed on mucosal surfaces (Ericson and Rundegren, 1983; Holmskov et al., 1997). It is found associated to alveolar macrophages as well as to epithelial cells of, e.g., the salivary glands, trachea, small intestine, and the genital tract (Holmskov et al., 1999; Mollenhauer et al., 2001; Bikker et al., 2002; Stoddard et al., 2007). Furthermore, gp340 is secreted into several body fluids such as saliva, lacrimal fluid, pancreatic juice, and bronchoalveolar secretions (Ericson and Rundegren, 1983; Holmskov et al., 1997; Schulz et al., 2002; Gronborg et al., 2004). Because of the discrepancies of names mentioned above we wish to suggest the name SALSA (salivary scavenger and agglutinin) for this protein based on its initial discovery in saliva and its function as a scavenger and agglutinin.

SALSA is a protein with 8–13 N-terminal SRCR domains, followed by two CUB-domains surrounding an additional SRCR domain. It thus belongs to the SRCR-protein family (Holmskov et al., 1999). Many members of this family are located on immune cells, and several of them have known functions as pattern recognition receptors (Resnick et al., 1994). SALSA was early on described as an agent that agglutinates *Streptococcus mutans*, but has since then been shown to bind a broad spectrum of microbes including Gram-positive and -negative bacteria as well as viruses (Ericson and Rundegren, 1983; Nagashunmugam et al., 1998; Prakobphol et al., 2000; Hartshorn et al., 2003; Bikker et al., 2004; Loimaranta et al., 2005; Leito et al., 2008). Part of the wide ligand binding capacity of SALSA is based on its ability to recognize conserved repeat motives on bacterial surface proteins (Loimaranta et al., 2009). A specific peptide sequence motif within the SRCR domains, VEVLXXXXW (X for any amino acid), has been shown to confer bacterial binding (Bikker et al., 2004).

SALSA has also been shown to interact with several endogenous ligands. Many of these are involved in innate immunity, such as surfactant proteins D and A (SP-D and SP-A), secretory IgA, trefoil factors, mucin-5B, lactoferrin, and complement factor C1q (Rundegren and Arnold, 1987; Boackle et al., 1993; Holmskov et al., 1997; Tino and Wright, 1999; Thim and Mortz, 2000; Thornton et al., 2001; Oho et al., 2004). The specific function of SALSA remains unknown. However, the identified ligands together with the localization of SALSA on mucosal surfaces suggest a role in the first-line immune defense, similar to that of other SRCR-family proteins.

The complement (C) system comprises more than 40 soluble and surface bound proteins (reviewed in Ricklin et al., 2010). It is organized into three activation pathways, the classical, the alternative, and the lectin pathway. The C system maintains a wide array of functions ranging from elimination of microorganisms, immune complexes and apoptotic cells to enhancing and directing adaptive immunity through opsonization and leukocyte chemotaxis. The three pathways are activated through distinct mechanisms. The classical pathway is activated via binding of C1q to targets, either directly or through antibodies or C-reactive protein (reviewed in Walport, 2001). The alternative pathway can be initiated spontaneously by hydrolysis and activation of C3 into active C3b. Subsequent activation depends on the absence of inhibitory structures from the target surfaces, which allows amplification of C3 cleavage to C3b (Walport, 2001). The lectin pathway is activated by binding of mannan-binding lectin (MBL) or ficolins to mannose, *N*-acetylglucosamine (GlcNAc), or other carbohydrate-containing structures on the surfaces of a wide array of target microbes, e.g., yeasts like *Candida albicans* and bacteria like *Staphylococcus aureus* and certain types of *Escherichia coli*. In the lectin pathway, MBL/ficolins associate with MBL-associated serine proteases 1 and 2 (MASP-1 and MASP-2). The MBL/MASP or ficolin/MASP complexes cleave C2 and C4 to generate the C4b2a complex, which in turn cleaves C3 into C3b on the surfaces of target microbes (reviewed in Thiel, 2007).

Although soluble complement components are present mainly in blood, they are also found in serous exudates on mucosal surfaces, such as in the oral cavity or in the airways (Boackle, 1991; Persson et al., 1991). This is particularly seen under pathological conditions, for example following mechanical damage or during infection, e.g., in periodontitis (Courts et al., 1977; Negut et al., 2007). When serous exudates filter through to the mucosal surfaces, soluble innate immune proteins bind to their targets and thereafter interact with mucosal surface receptors. This creates a specific interplay of defense mechanisms against invading microorganisms. Our search for novel functions of SALSA led to the identification of an interaction with the C lectin pathway. In this study the aim was to investigate how SALSA interacts with the lectin pathway to control C activation on the mucosal surfaces.

Our results show that SALSA has a dual role in regulating the lectin pathway. It acts as an activator on surfaces and as an inhibitor in the fluid phase. This suggests that SALSA could target the innate immune responses to certain microorganisms while simultaneously limiting inflammation in the fluid phase.

## MATERIALS AND METHODS

### PROTEINS, ANTIBODIES, AND SERA

SALSA was purified from human parotid saliva by bacterial binding and EDTA-elution as described previously (Prakobphol et al., 2000). Recombinant SALSA (rSALSA) was expressed in Chinese hamster ovary cells using a vector system (End et al., 2005) and purified as above. Recombinant MBL (rMBL; Jensenius et al., 2003), recombinant M-ficolin (rM-ficolin; Wittenborn et al., 2010), plasma purified L-ficolin and H-ficolin (Matsushita et al., 2000; Zacho et al., 2012), recombinant MASP-2 (rMASP-2; Thiel et al., 2009), plasma purified C4 (Dodds, 1993), and plasma purified C3 (Tack and Prahl, 1976) were obtained as described previously. C1q was purchased from Quidel (San Diego, CA, USA).

Mouse monoclonal anti-M-ficolin (7G1) was produced as described previously (Wittenborn et al., 2010). Mouse monoclonal anti-MBL (Hyb 131-01) and anti-SALSA (Hyb 213-06) antibodies were from Bioporto, Denmark. Mouse monoclonal anti-H-ficolin (4H5) and rat monoclonal anti-MASP-2 (8B5) were obtained from Hycult Biotechnology, The Netherlands. Rabbit anti-C3c, anti-C4c, and anti-C1q antibodies were purchased from Dako, Denmark. HRP-conjugated rabbit anti-mouse IgG and goat anti-rabbit IgG antibodies were from Jackson ImmunoResearch Laboratories (West Grove, PA, USA). Alexa 488-coupled goat anti-mouse IgG and goat anti-rabbit IgG antibodies were obtained from Invitrogen (Carlsbad, CA, USA).

Normal human serum (NHS) was taken from healthy volunteers with written informed consent, pooled, and aliquoted for storage at -70°C. MBL-deficient serum was obtained from a person known to lack MBL by screening individual sera from healthy volunteers. To block the classical and lectin pathways of complement, EGTA with MgCl_2_ (in final concentrations of 10 mM and 5 mM, respectively) was added to NHS (MgEGTA-serum). Heat-inactivated serum (HIS) was made by incubating a sample from the above described serum pool at 56°C for 30 min.

### MEASUREMENT OF COMPLEMENT ACTIVATION

The effect of SALSA in the fluid phase on the three activation pathways of complement was tested using the Wieslab^®^ Complement System Screen ELISA assay (Euro-Diagnostica, Sweden). This assay measures how SALSA affects the activation of complement on surfaces coated with pathway-specific activators. Deposition of C5b-9 in the wells is measured as the end-point. SALSA was diluted in serum at concentrations of 0, 1.0, 3.0, and 10 μg/ml, and the samples were subsequently added to ELISA wells coated with specific activators for the three different complement pathways. The lectin pathway activation was additionally investigated using SALSA at concentrations of 0.003, 0.03, and 0.3 μg/ml. Activation of complement was measured as generation of the terminal C complex onto the activating surfaces according to the manufacturer’s instructions.

### ELISA BINDING ASSAYS

For SALSA binding studies rMBL, rM-ficolin, L-ficolin, H-ficolin, C1q, C4, and C3 (each 1 μg/ml) in coating buffer (15 mM Na_2_CO_3_, 35 mM NaHCO_3_, pH 9.6) were coated onto a Maxisorp plate (Nunc, Denmark). Additional binding sites on the plate were blocked with 5% non-fat milk in Tris-buffered saline (TBS; 140 mM NaCl, 5 mM Tris, pH 7.4) containing 1 mM Ca^2+^ and 0.05% Tween-20 (TBS/Ca/Tween). The Ca^2+^-dependency of the binding was investigated by omitting Ca^2+^ from the buffer and having 10 mM EDTA added instead. Thus, the wells were washed with either TBS/Ca/Tween or TBS/EDTA/Tween. rSALSA (0.5 μg/ml) in TBS/Ca or TBS/EDTA was added and incubated for 1 h at 37°C. Binding was detected using anti-SALSA (0.1 μg/ml) and HRP-conjugated rabbit anti-mouse antibodies (1:10,000 in TBS/Ca or TBS/EDTA). OPD tablets (Dako) were used for development and the color reaction was measured with an iEMS Reader MF (Labsystems, Espoo, Finland) at an OD of 492 nm.

### ELISA COMPETITION ASSAYS

The effect of monosaccharides on MBL binding to solid phase SALSA was tested in an ELISA assay. For this 0.1 μg/ml of rSALSA or 10 μg/ml of mannan (Sigma, St. Louis, MO, USA) were used for coating onto wells of Maxisorp plates as described above. rMBL at 1 μg/ml was mixed in the fluid phase with mannose, GlcNAc, or glucose (all from Sigma) in concentrations ranging between 0 and 100 mM in TBS/Ca. The samples were added to the plate and incubated for 1 h at 37°C. Binding was detected with anti-MBL (0.1 μg/ml) followed by HRP-conjugated rabbit anti-mouse IgG antibodies (1:10,000 in TBS/Ca).

The effect of fluid-phase SALSA on the binding of the MBL/MASP-2 complex to mannan was tested in an ELISA assay. Mannan (10 μg/ml) was coated in Maxisorp wells. rMBL (0.5 μg/ml) was mixed with rMASP-2 (0.1 μg/ml) in TBS/Ca and rSALSA was added in final concentrations of 0, 0.05, 0.15, 0.5, and 1.5 μg/ml. Subsequently, the samples were incubated for 1 h at 37°C on the mannan-coated plate. Binding was detected with anti-MBL or anti-MASP-2 (both 0.1 μg/ml) and HRP-conjugated rabbit anti-mouse IgG antibodies (1:10,000 in TBS/Ca).

### INHIBITION OF MBL BINDING TO MICROORGANISMS BY SALSA

The effect of SALSA on the binding of MBL to *C. albicans *and *E. coli* was studied in a flow cytometry assay. *C. albicans* and *E. coli *were clinical blood culture isolates from the Helsinki University Central Hospital Laboratory (HUSLAB). Both strains were identified using routine microbiological techniques. *C. albicans* was grown in yeast-extract peptone dextrose medium overnight at 30°C with shaking. *E. coli* was grown in LB medium overnight at 37°C with shaking. Both microbes were washed with veronal-buffered saline (VBS; 142 mM NaCl, 5.0 mM sodium barbital, pH 7.4) containing 1 mM Ca^2+^ (VBS/Ca) by centrifugation for 5 min at 1,000 ×*g*. *C. albicans* was resuspended to 5 × 10^7^ cells/ml and *E. coli* was resuspended to 2.4 × 10^8^ cells/ml. Volumes of 100 μl of these dilutions were used for each sample. rMBL (0.9 μg/ml, which corresponds to 20% of the Finnish average serum MBL level; Aittoniemi et al., 1996) was mixed with rSALSA in final concentrations of 0, 0.05, 0.15, 0.5, 1.5, and 4.5 μg/ml. The proteins were incubated with the microbes for 30 min at 37°C with shaking. After washing with VBS/Ca anti-MBL antibody (5 μg/ml in VBS/Ca) was added and incubated for 30 min at 37°C. Alexa 488-coupled goat-anti-mouse IgG antibody (1:400 in VBS/Ca) was used as a secondary antibody. The microbes were fixed in 1% paraformaldehyde and analyzed by CyAn ADP (Dako). Forward and sideward scatters were used to define the cell population and 10,000 events were routinely counted. The mean fluorescence intensity (MFI) values were used for quantification of the data.

### SALSA-MEDIATED COMPLEMENT ACTIVATION ASSAY

The ability of surface-coated SALSA to interfere with C4 and C3 deposition was tested in an ELISA assay. Mannan (10 μg/ml) and rSALSA (0.5 μg/ml) were coated on a Maxisorp plate as described above. NHS, MBL-deficient serum, MgEGTA-serum, and HIS were diluted 1:10 in TBS/Ca and incubated on the plates for 30 min at 37°C. Complement C4 and C3 deposition was detected by incubation with polyclonal anti-C4c and C3c antibodies (1:5000) for 1 h at 37°C, followed by HRP-conjugated goat anti-rabbit antibodies (1:10,000 in TBS/Ca). The enzyme reaction was developed as described above.

The complement regulating property of SALSA was also tested using flow cytometry. *C. albicans* was grown as described above. rSALSA was diluted in 10% NHS, MBL-deficient serum, MgEGTA-serum, or HIS. The final rSALSA concentrations were 0, 0.05, 0.15, 0.5, and 1.5 μg/ml. The serum samples were incubated with *C. albicans* for 30 min at 37°C. C4 and C3 deposition was measured using anti-C4c and C3c antibodies (1:200 in VBS/Ca) followed by detection using Alexa 488-conjugated goat-anti-rabbit IgG antibody (1:400). The yeast cells were fixed in 1% paraformaldehyde and analyzed by CyAn ADP as described above.

### STATISTICAL ANALYSIS

Student’s paired, two-tailed *t*-test was used to calculate statistical significance of complement activation mediated by surface-coated SALSA.

## RESULTS

### THE EFFECT OF FLUID-PHASE SALSA ON COMPLEMENT ACTIVATION

**FIGURE 1:**
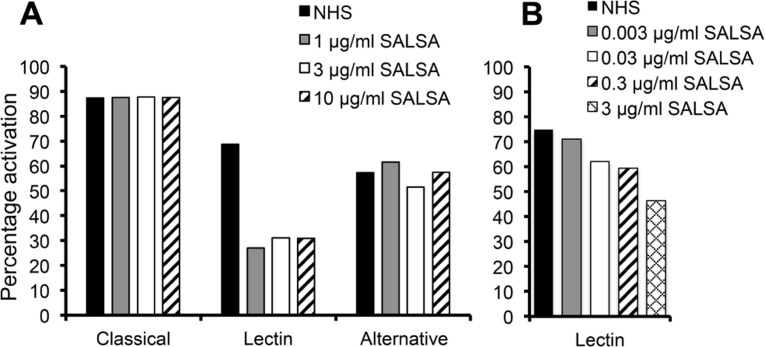
**Inhibition of complement activation by fluid-phase SALSA.** In an ELISA assay (Wieslab^®^) SALSA (0–10 μg/ml) was diluted in NHS and the activation of the classical, the lectin, and the alternative pathway of complement was measured. Inhibition of the lectin pathway but not the classical or the alternative pathways was observed when SALSA was added **(A)**. The effect on the lectin pathway was investigated with further dilutions of SALSA. This revealed a clear dose-dependency **(B)**. Data from single experiments performed in duplicate are shown. The results are expressed as percentage of activation in a standardized control serum pool supplied by the manufacturer.

The complement regulating properties of SALSA were initially studied in an ELISA assay separating the three distinct pathways of complement activation: the classical, the lectin, and the alternative pathway. Complement activation was measured by C5b-9 deposition in the absence or presence of purified SALSA. The presence of 1 μg/ml of SALSA in serum inhibited the lectin pathway by 57% but had no effect on the classical or the alternative pathway (**Figure 1A**). The effect of SALSA on the lectin pathway was investigated in greater detail using lower concentrations (**Figure 1B**). This revealed a clear dose-dependent inhibition of the lectin pathway when SALSA was in the fluid phase.

### BINDING OF SALSA TO COMPLEMENT COMPONENTS

**FIGURE 2:**
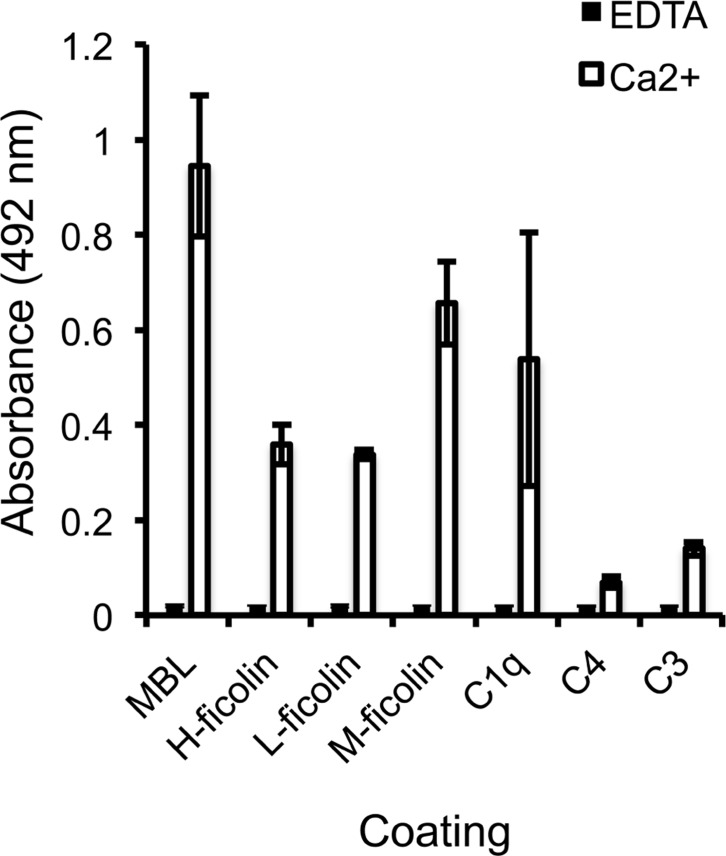
**Interaction of SALSA with complement proteins.** MBL, M-ficolin, L-ficolin, H-ficolin, C1q, C4, and C3 (1 μg/ml) were coated on a plate followed by incubation with rSALSA (0.5 μg/ml) in either a Ca^2+^- or EDTA-containing buffer. SALSA bound strongly to MBL, but also binding to all three ficolins and C1q was observed. Only a weak interaction with C4 and C3 could be seen. In the presence of EDTA, no binding of SALSA occurred. The averages and standard deviations (SDs) of two experiments performed in duplicate are shown.

To identify the specific interactions involved in SALSA-mediated inhibition of the lectin pathway, an ELISA assay was used. MBL, H-, L-, and M-ficolin, C1q, C4, and C3 were coated on a plate and varying concentrations of rSALSA were added with or without Ca^2+^. Previous binding of SALSA to the classical pathway component C1q has been observed (Boackle et al., 1993) and this was added as a positive control (**Figure 2**). We observed a strong binding of SALSA to MBL, and also some binding to all three ficolins and C1q. Only a weak binding was seen to C4 and C3. Ca^2+^-depletion by EDTA abolished all of the protein–protein interactions. Similar results were obtained using purified SALSA instead of recombinant SALSA (data not shown).

### INHIBITION OF THE MBL–SALSA INTERACTION BY CARBOHYDRATES

**FIGURE 3:**
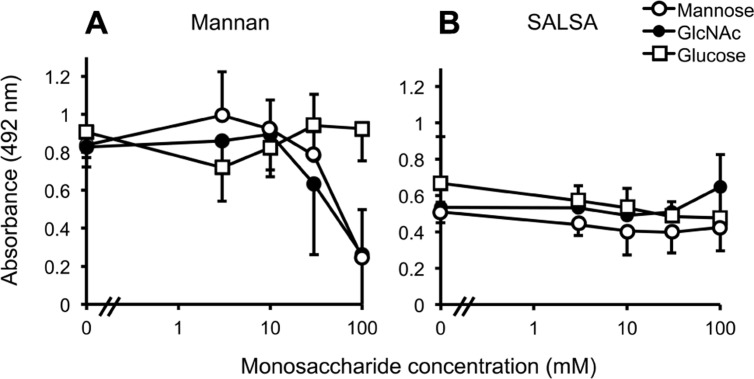
**The effect of monosaccharides on MBL binding to mannan and SALSA.** In an ELISA assay mannan **(A)** or rSALSA **(B)** were coated on a microtiter plate. rMBL (1 μg/ml) was mixed with mannose, GlcNAc, or glucose (all 0–100 mM). Binding was detected with anti-MBL antibody. The presence of mannose and GlcNAc inhibited dose-dependently the binding of MBL to mannan while glucose had no effect **(A)**. In contrast, the binding of MBL to SALSA was not inhibited even by high concentrations of the saccharides **(B)**. Averages and SDs of three experiments performed in duplicate are shown.

In order to pinpoint whether SALSA binds to the carbohydrate recognition site in MBL, the MBL–SALSA interaction was compared with the MBL–mannan interaction. For this purpose, mannan and rSALSA were coated in wells and binding of MBL in the presence of varying concentrations of mannose, GlcNAc and glucose was tested. Mannose and GlcNAc are known carbohydrate ligands for the carbohydrate recognition domain (CRD) part of MBL, whereas glucose shows a much weaker interaction. As expected, both mannose and GlcNAc inhibited the binding of MBL to mannan, whereas glucose had no effect (**Figure 3A**). When rSALSA was coated on a plate and the MBL–carbohydrate mixtures were added the presence of mannose, GlcNAc, or glucose had no effect, even at 100 mM concentration, on the binding of MBL to SALSA (**Figure 3B**). Similar results were obtained when purified SALSA was used instead of recombinant SALSA (data not shown).

### THE EFFECT OF SALSA ON THE MBL–MANNAN INTERACTION

**FIGURE 4:**
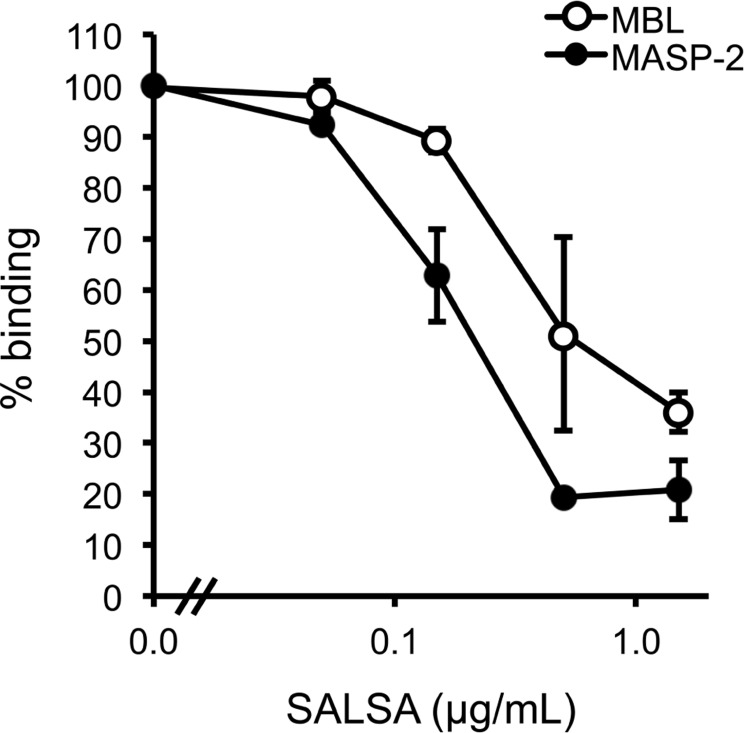
**The effect of fluid-phase SALSA on the binding of the MBL/MASP-2 complex to mannan.** In an ELISA assay mannan was coated in microtiter wells. rMBL was mixed with rMASP-2 and varying amounts of rSALSA were added. Binding was detected with an anti-MBL antibody. A dose-dependent inhibition of MBL binding by rSALSA was observed. As a control, a similar analysis using an anti-MASP-2 antibody confirmed the inhibition. The results are expressed as percentage of MBL or MASP-2 binding in the absence of SALSA. Averages and SDs of three experiments performed in duplicate are shown.

Next, we wanted to test whether SALSA would interfere with the MBL–mannan interaction. In an ELISA assay MBL was mixed with MASP-2 and varying concentrations of rSALSA and then added to a mannan-coated microtiter plate. The binding of MBL to the mannan-coated surface was measured using monoclonal anti-MBL and anti-MASP-2 antibodies (**Figure 4**). MBL forms a complex with MASP-2 under physiological conditions. Therefore, we also measured the binding of MASP-2 to verify the result. A clear binding of both MBL and MASP-2 to the mannan surface was observed, suggesting the formation of the MBL/MASP-2 complex. When increasing amounts of rSALSA were added, a dose-dependent decrease of both MBL and MASP-2 binding to mannan was seen. The slight difference seen in the absorbance of MBL and MASP-2 is due to the ELISA technique and the different antibodies used for detection. The similar inhibition tendency suggests that both the MBL and MASP-2 measurements represent the binding of the whole complex to mannan.

### SALSA INHIBITS THE BINDING OF MBL TO *C. ALBICANS* AND *E. COLI*

**FIGURE 5:**
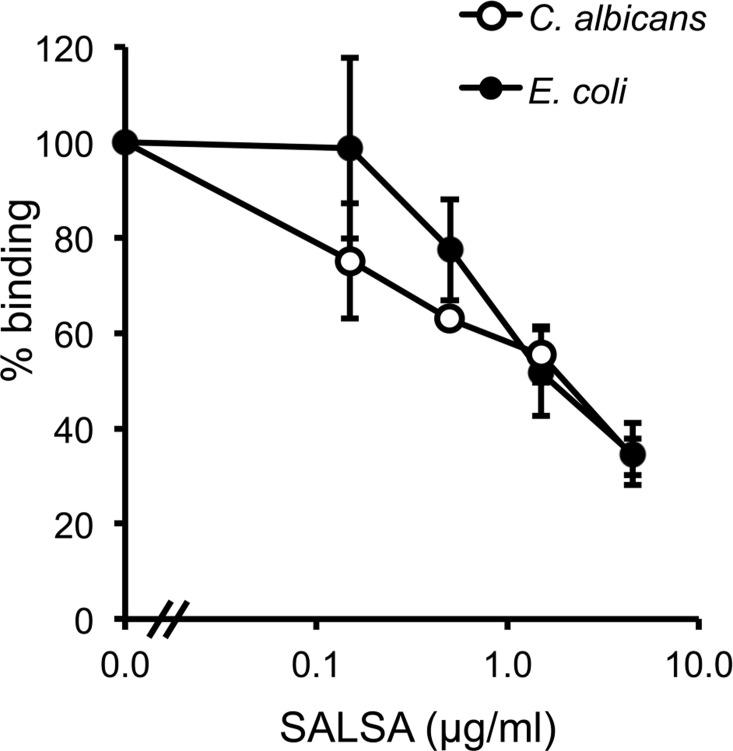
**The effect of SALSA on the binding of MBL to its microbial targets *C. albicans* and *E. coli*.** In a flow cytometry assay *C. albicans* and *E. coli* were incubated with a mixture of rMBL (0.9 μg/ml) and rSALSA (0–45 μg/ml). Binding of MBL was detected with an anti-MBL antibody. The binding of MBL to *C. albicans* and *E. coli* decreased dose-dependently when SALSA was present. The results are expressed as percentage of MFI in the absence of SALSA. Averages and SDs of three separate experiments are shown.

To verify the above observation using a more physiological approach, a similar assay was performed using known microbial targets of MBL. The binding of MBL to *C. albicans* and *E. coli* in the presence of SALSA was tested in a flow cytometry assay. rSALSA and rMBL were mixed and then incubated with the microbes. MBL binding was detected using a monoclonal anti-MBL antibody (**Figure 5**). Binding of MBL in the absence of rSALSA was observed both to *C. albicans* and *E. coli* with average MFIs of 18.5 and 31.0, respectively. However, the addition of rSALSA inhibited the binding of MBL to *C. albicans* and *E. coli* dose-dependently. Since our carbohydrate inhibition assay showed that GlcNAc and mannose inhibit the binding of MBL to mannan, we wanted to rule out an inhibitory effect of a possible sugar contamination in the SALSA preparation. Therefore, a similar experiment was done using heat-treated SALSA. Boiling for 5 min destroys the protein but would not affect carbohydrates. Boiled SALSA (0.1–45 μg/ml) had no inhibitory effect on the binding of MBL to *C. albicans* (data not shown).

### SALSA MEDIATES COMPLEMENT ACTIVATION WHEN BOUND TO A SURFACE

**FIGURE 6:**
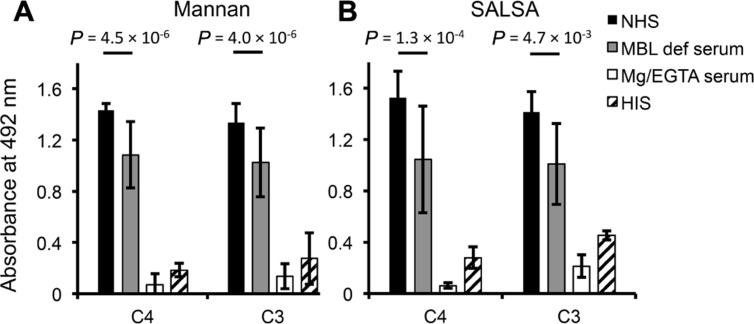
**Complement activation by surface-bound SALSA.** In an ELISA assay mannan **(A)** or rSALSA **(B)** were coated in microtiter wells. NHS, MBL-deficient serum, MgEGTA-serum, or HIS (all 10%) was added and C4 and C3 deposition was measured using specific antibodies. Averages and SDs of three experiments performed in duplicate are shown.

To see the effect of surface-coated SALSA on C activation rSALSA was coated on a microtiter plate and C4 and C3 deposition were measured after incubation of 10% NHS, MBL-deficient serum, MgEGTA-serum, or HIS. A parallel experiment was done using a mannan-coated plate. On both SALSA- and mannan-coated plates deposition of C4 and C3 was seen from NHS (**Figures 6A, B**). C4 and C3 deposition was reduced when MBL-deficient serum was used. The C4 deposition, read as absorbance, to SALSA and mannan from MBL-deficient serum was decreased when compared with NHS by 32 and 24%, respectively. For C3 the similar absorbance values decreased by 29 and 23%, respectively. Thus, a substantial amount of C4 and C3 remained bound. When MgEGTA-serum was used C4 and C3 deposition to both SALSA and mannan was almost completely abolished which verifies that the observed activation is due to classical and lectin pathway activation. When HIS was used some deposition of C4 and C3 to both SALSA and mannan could still be seen. The loss of this direct interaction when MgEGTA serum was used is in line with the direct C4-SALSA and C3-SALSA binding assay (**Figure 2**). Thus we observed MBL-mediated complement activation as measured by C4 and C3 deposition to surface-coated SALSA.

### SALSA INHIBITS COMPLEMENT ACTIVATION IN THE FLUID PHASE

The flow cytometry assay using *E. coli* and *C. albicans* showed that SALSA in the fluid phase inhibits the binding of MBL to microbes (see above). To measure the effect of this inhibition on the overall complement activation against microbes, we used a flow cytometry assay measuring C4 and C3 deposition on *C. albicans* in the presence of varying concentrations of SALSA.

**FIGURE 7:**
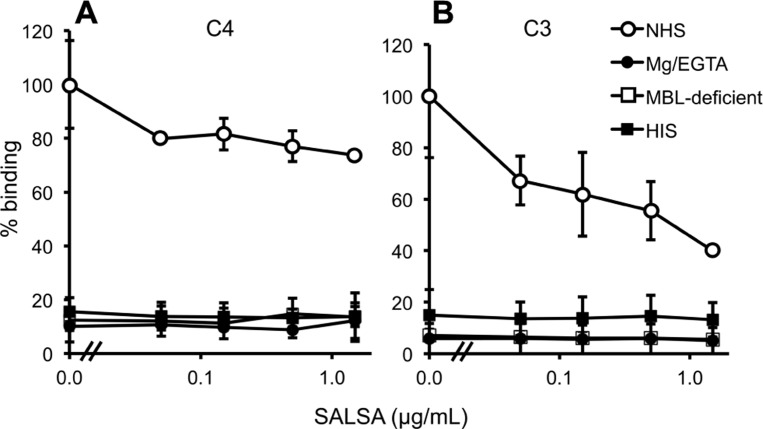
**Complement inhibition by fluid-phase SALSA.** In a flow cytometry assay *C. albicans* was incubated in 10% NHS, MBL-deficient serum, MgEGTA-serum, or HIS with rSALSA (0–15 μg/ml). Complement deposition was measured using anti-C4 **(A)** and anti-C3 **(B)** antibodies. In **(A)** an inhibition of C4 deposition was observed when SALSA was added to NHS. **(B)** shows a dose-dependent inhibition of C3 deposition from NHS. The results are expressed as percentage of MFI in the absence of SALSA. Averages and SDs of three separate experiments are shown.

Normal human serum, MBL-deficient serum, MgEGTA-serum, or HIS (all at 10%) was used. C4 deposition to *C. albicans* was observed from NHS (**Figure 7A**). When using MBL-deficient serum, MgEGTA-serum, or HIS no C4 deposition was observed. The effect of adding varying concentrations of rSALSA showed a weak inhibition of the C4 deposition from an initial average MFI of 20.7–15.2. In the case of C3 (**Figure 7B**) a small amount of C3 deposition was still observed when HIS was used, but no deposition was observed when MBL-deficient or MgEGTA-sera were used. Using NHS a clear C3 deposition was observed. When rSALSA was added a dose-dependent decrease of C3 deposition was seen from an initial average MFI of 72.6–29.0. The experiments were repeated using 20% serum (data not shown), giving similar results. These data suggest that the presence of SALSA in the fluid phase inhibits the activation of MBL-mediated complement activation and subsequent deposition of complement factors C4 and C3 on *C. albicans*.

## DISCUSSION

In this report we have been studying the interplay between SALSA and the complement system. We have especially concentrated on the effect of SALSA on complement activation and how this is mediated. We observed that SALSA can bind directly to MBL. This interaction has a dual effect on MBL-mediated complement activation. SALSA bound to a surface may lead to activation of the lectin pathway of complement. However, when SALSA is in the fluid phase, lectin pathway activation can be inhibited.

Using an ELISA assay measuring complement activation we initially observed a specific SALSA-mediated inhibition of the lectin pathway (Reichhardt et al., 2011). SALSA has been described to bind to a wide range of both endogenous and microbial ligands (reviewed in Madsen et al., 2010). In order to understand the underlying mechanism of the lectin pathway inhibition we investigated SALSA interactions with complement components. We found that SALSA bound to surface-coated MBL, H-ficolin, L-ficolin, M-ficolin, and C1q. Binding to these complement components was also observed in the reverse configuration with surface-bound SALSA (data not shown). These binding interactions were tested with both recombinant and purified SALSA with similar results (data not shown). All the observed interactions were Ca^2+^-dependent. Together with the already known SALSA ligands, SP-D and SP-A, these proteins form a group of structurally very similar soluble proteins functioning as pattern recognition receptors. They all resemble bouquets of flowers comprising N-terminal collagen-like “stalks” linked to C-terminal globular “head” domains, CRD for MBL, SP-A, and SP-D, fibrinogen-like recognition domains for the ficolins and globular region for C1q (reviewed in Holmskov et al., 2003; Wallis et al., 2010). The interactions between MBL/ficolins/C1q and their respective associated serine proteases, MASPs and C1r/C1s, are also known to be calcium-dependent. In the case of the serine proteases the calcium-dependency of the binding is mediated through an epidermal growth factor-like module, which is embedded between two CUB domains (Feinberg et al., 2003; Gregory et al., 2003). SALSA contains two similar CUB domains surrounding a SRCR domain. Thus, it is likely that the calcium-dependency of the binding is mediated through this site.

Since SALSA bound to MBL and vice versa, we proceeded to investigate the effect of SALSA on MBL binding to its known monosaccharide ligands mannose and GlcNAc and to the yeast surface structure mannan. Even 100 mM concentrations of mannose or GlcNAc did not inhibit the binding of MBL to surface-coated SALSA. In the control setting, 100 mM mannose and GlcNAc inhibited the binding of MBL to mannan, as expected. Based on these data, the binding site for SALSA on MBL would be different from the binding site for carbohydrates in the CRD. However, when SALSA was mixed in fluid phase with MBL and MASP-2, SALSA inhibited the binding of the MBL–MASP-complex to surface-coated mannan dose-dependently. When MBL was mixed with mannose before addition to the SALSA-coated plate, SALSA was still able to bind to MBL. MBL does therefore not appear to utilize the same site for binding to SALSA and mannan. However, when MBL/MASP-2 interacted with SALSA prior to exposure to mannan, the binding was inhibited. This suggests that although the binding site is different, the binding of SALSA influences the interaction between MBL and mannan. This could either be through steric hindrance by the big SALSA molecule (340 kDa) or by an impact of SALSA on the tertiary structure of MBL.

The function of MBL depends on its recognition of microbial targets, such as *C. albicans* or *E. coli* (reviewed in Jack and Turner, 2003). To further elucidate the physiological consequences of SALSA binding to MBL, we investigated the effect of SALSA on MBL binding to these microbial targets. Binding of SALSA to both *E. coli* and *C. albicans* was tested using flow cytometry but no binding was observed (data not shown). However, we observed a dose-dependent inhibition of MBL binding to both *C. albicans* and *E. coli*, suggesting that SALSA binds MBL in solution and blocks the interaction of MBL with the microbes. The physiological concentration of SALSA in saliva has been estimated to be 0.5 μg/ml (Hartshorn et al., 2003). At this concentration an inhibition was observed. Thus, MBL can bind to certain microbes and activate complement but in the case of, e.g., *C. albicans*, SALSA inhibits MBL binding and activation of the lectin pathway.

The interaction between C1q and SALSA has previously been suggested to be sufficient to initiate complement activation through the classical pathway. This was observed by utilizing an assay measuring disappearance of native C4 and appearance of C4b in a Western blot assay (Boackle et al., 1993). Another study showed activation of complement through the lectin pathway (Leito et al., 2011). Since we had initially observed inhibition of the lectin pathway we now investigated the overall effect of SALSA on complement activation. The deposition of C4 and C3 was measured in an ELISA assay with surface-bound SALSA. By comparing NHS, MBL-deficient serum (where no MBL-mediated complement activation occurs), Mg/EGTA serum (where no classical or lectin pathway activation occurs), and HIS (where no complement activation at all occurs) the amount of complement deposition mediated by each distinct pathway could be elucidated. In the solid-phase assay the use of SALSA-coated plates and mannan-coated plates provided a platform for comparing the effects of SALSA to a known lectin pathway activator. The clear deposition of C4 and C3 from NHS on both plates showed that SALSA as well as mannan on the solid phase was able to activate the complement system. Our results suggested that several mechanisms are involved, including activation mediated by MBL. In line with others (Leito et al., 2011) we observed a difference in the C4 and C3 deposition when MBL-deficient serum was used. Approximately 30% of the total complement activation was based on the presence of MBL, confirming the relevance of our observed interaction between SALSA and MBL. However, substantial C activation occurred even when MBL-deficient serum was used. This supports the previously described SALSA-mediated C activation through C1q and the classical pathway (Boackle et al., 1993). Others have previously tried to block this residual activation by utilizing specific classical pathway inhibiting antibodies but they were unable to block all the C4 deposition (Leito et al., 2011). The interaction of SALSA with all the three ficolins that we observed could be the reason for this residual activation. A substantial amount of C4 and C3 binding was observed from HIS. This correlates with the weak calcium-dependent interaction between SALSA and C4 and C3, and could be another explanation for the residual complement deposition. However, the appearance of similar C4 and C3 deposition on the mannan-coated surface implies that this simply could represent the background level of binding in the assay. The loss of activation in Mg/EGTA serum supports our earlier finding of the Ca^2+^-dependency of the interactions between SALSA and MBL, ficolins and C1q. However, part of the lower level of complement activation must be accredited to the Ca^2+^-dependency of the C1qrs and MBL/ficolin–MASP complexes. In conclusion, we suggest that surface-bound SALSA acts as a complement activator through the classical and lectin pathways via direct interactions with C1q, MBL, and the ficolins.

In our initial experiment we observed inhibition of the lectin pathway by fluid-phase SALSA but no effect on the classical or alternative pathway activation. However, we also confirmed the SALSA-mediated classical and lectin pathway activation as described previously (Boackle et al., 1993; Leito et al., 2011). With SALSA in the fluid phase the classical pathway is initiated by C1q bound to targets, for example to LPS or surface-bound antibodies and SALSA has no effect. Boackle et al. (1993) showed the ability of surface-coated SALSA to interact with C1q directly. This was also confirmed by our assays with surface-bound SALSA (**Figures 2 and 6**). Also, Leito et al. (2011) measured the effect of SALSA coated to a surface and showed that it activates the lectin pathway. This was also confirmed by our assay with surface-coated SALSA. However, fluid-phase SALSA was capable of preventing MBL binding to the surface carbohydrates and thus inhibiting lectin pathway activation. Thus, complement activation or inhibition by SALSA seems to depend on whether SALSA is on the surface or in the fluid phase, respectively.

In order to understand the role of fluid-phase SALSA we investigated its effect on complement activation by measuring C4 and C3 deposition on *C. albicans* by flow cytometry. We observed an inhibition of C4 and C3 deposition from NHS on *C. albicans* by SALSA. The lack of classical pathway activity in the MBL-deficient serum activation suggests that this serum does not have antibodies against *C. albicans*. SALSA thus inhibits MBL binding to *C. albicans* and the end effect is decreased complement activation. This result verifies that soluble SALSA could function as a physiologically relevant inhibitor of the lectin pathway of complement. Inhibition is mediated through an inhibition of MBL binding and leading to decreased C4 and C3 deposition on the surface of *C. albicans*.

**FIGURE 8:**
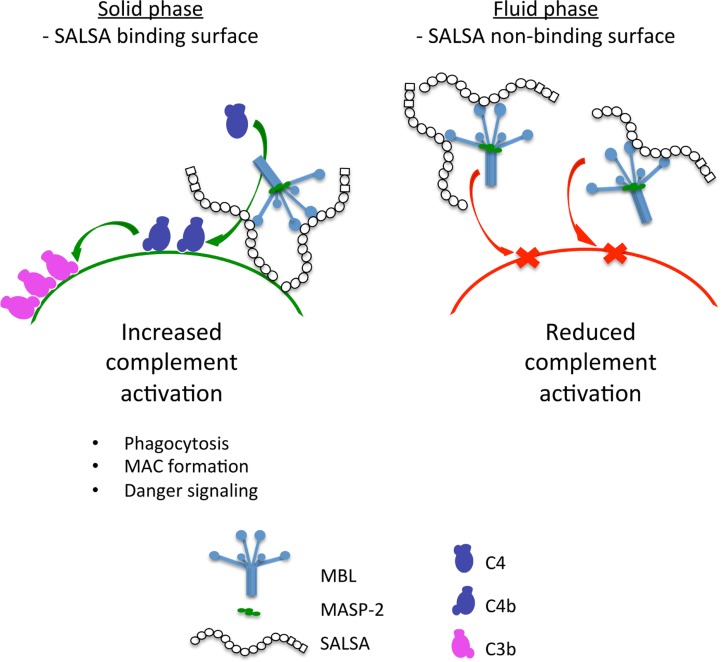
**A graphical presentation of the dual function of SALSA.** Binding of SALSA to a surface allows recruitment of MBL, C1q, and ficolins and subsequent complement activation. In contrast, soluble SALSA interacts with MBL in the fluid phase drawing it away from the surface. Consequently, the lectin pathway of complement is inhibited.

The experimental work presented here shows that soluble SALSA is a novel regulator of the lectin pathway of complement. However, SALSA appears to have a dual physiological role. We find that SALSA acts as an activator of complement when it is bound to a surface. In contrast, when SALSA is free in the fluid phase it acts as an inhibitor of the lectin pathway of complement. The direct binding to MBL, C1q and possibly ficolins may account for the C activating effects. SALSA has been shown to mediate aggregation of bacteria. It has been suggested that the repeating structure of SALSA may enable the protein to interact with several ligands at the same time (Edwards et al., 2008). The lectin pathway inhibition is likely due to formation of similar soluble complexes with MBL. This hypothesis is depicted in **Figure 8**.

It has previously been shown that the binding properties of SALSA differ depending on whether the protein is bound to a surface or is free in the fluid phase. The fluid-phase and surface-adsorbed SALSA displayed different aggregating and adhering potential toward bacteria. It was speculated that this disparity correlated with the pathogenicity of the strains (Loimaranta et al., 2005). The fact that SALSA does not bind to *C. albicans*, but inhibits complement in the fluid phase could, in fact, be one of the mechanisms underlying the property of *C. albicans* to commonly cause infections in the oral and other body cavities.

The targeting of SALSA to a certain surface, e.g., to a microbe, would allow SALSA to assist in the recruitment of MBL, C1q and ficolins to the surface and subsequently enable complement activation on the surface of the microbe. In contrast, when SALSA is free in the fluid phase it interacts with MBL and possibly C1q and ficolins. This interaction could lead to protein aggregation thus keeping the complement initiators from interacting with their targets and in doing so inhibiting the complement response and subsequent inflammation. On the other hand, suppression of C activation would help microbes, e.g., those in the “normal flora” or *C. albicans* to escape C attack.

## Conflict of Interest Statement

The authors declare that the research was conducted in the absence of any commercial or financial relationships that could be construed as a potential conflict of interest.
